# Changes in Resting-State Cerebral Activity in Women With Polycystic Ovary Syndrome: A Functional MR Imaging Study

**DOI:** 10.3389/fendo.2020.603279

**Published:** 2020-12-10

**Authors:** Guanghui Li, Junhao Hu, Si Zhang, Weijie Fan, Li Wen, Guangxian Wang, Dong Zhang

**Affiliations:** Department of Radiology, XinQiao Hospital, Army Medical University, ChongQing, China

**Keywords:** polycystic ovary syndrome, fasting insulin, amplitude of low-frequency fluctuation, functional connectivity, resting-state functional magnetic resonance imaging, insulin resistance, luteinizing hormone

## Abstract

**Background:**

Previous studies have found that women with polycystic ovary syndrome (PCOS) have some degree of brain function change as well as cognitive function and emotions, such as poor executive functioning and memory, anxiety and depressive symptoms. However, the neurobiological mechanisms underlying these alterations have not yet been clarified.

**Method:**

Fasting serum hormone testing, neuropsychological testing and resting-state magnetic resonance imaging (rs-fMRI) were performed in 41 women with newly diagnosed PCOS and 41 healthy controls matched by age and education during their 2–5 days of menstrual period. Analysis of the amplitude of low-frequency fluctuation (ALFF) was used to calculate the seed points. Then, the functional connectivity (FC) values between these abnormal seed points and other voxels in the whole brain were calculated. Finally, the correlations among clinical indexes, neuropsychological evaluation scores, and neuroimaging data were analyzed.

**Results:**

Compared with the control group, the PCOS group showed higher luteinizing hormone (LH) and serum insulin levels, worse sleep quality, increased depressive and anxiety state scores, and memory and executive function impairments. Pearson’s correlation analysis showed that the decreased ALFF value in the left middle frontal gyrus (MFG.L), which is related to poor executive performance and depressive disorders, was negatively correlated with the plasma insulin level in subjects with insulin resistance. Furthermore, the increased FC strength between the MFG.L and left inferior frontal gyrus (IFG.L) was positively correlated with the serum testosterone level. The enhanced FC strength between the left posterior cingulate gyrus (PCG.L) and triangular part of the left inferior frontal gyrus (IFGtriang.L) was negatively correlated with the plasma LH level. When use the right middle occipital gyrus (MOG.R) as the seed point, the FC strength with the right inferior occipital gyrus (IOG.R), which is associated with impaired memory, was decreased.

**Conclusion:**

The ALFF and FC results elucidated brain functional abnormalities at the regional and network levels in women with PCOS, while correlation analyses simultaneously demonstrated that these alterations were associated with serum hormones and cognitive function. These results may provide useful information regarding the potential mechanisms of cognitive impairment and emotional changes in this population.

## Introduction

Polycystic ovary syndrome (PCOS) is one of the most common endocrine and metabolic disorders among women of childbearing age, with a high prevalence of 5–15% during this stage of life according to the Rotterdam criteria (1–5). PCOS is characterized by clinical and/or biochemical hyperandrogenism, ovulatory dysfunction, and polycystic ovaries (5). In addition, PCOS is often accompanied by obesity, obstructive sleep apnea, insulin resistance (IR), abnormal glucose tolerance, lipid metabolic disorders, and fatty liver disease (6, 7). These symptoms not only affect reproductive function but also increase the risk of type 2 diabetes mellitus (T2DM), cardiovascular disease, metabolic syndrome and endometrial cancer during the middle of the patients’ lifespan or later in life (8, 9). The etiology of PCOS is still not completely understood, but the current evidence suggests that the syndrome may result from genetic susceptibility interacting with environmental risk factors, including diet and lifestyle factors (6, 7, 10, 11). IR and hyperinsulinemia may play an important role in metabolic disorders, independent of but exacerbated by obesity (6, 12).

Previous studies showed that compared with healthy controls, women with PCOS more commonly suffered from mood disorders, such as anxiety and depression, and sleep disturbances were also very common among the patients (13–17). Some studies have indicated that the cognitive function of women with PCOS is partly impaired due to abnormal hormone levels, such as testosterone (T) (18, 19) and fasting insulin (FINS) (20, 21). Diffusion tensor magnetic resonance imaging (DTI) studies also showed that the white matter (WM) microstructure between PCOS and control groups was different and that compared with controls, the patient group had a poor cognitive performance (22). A functional magnetic resonance imaging (fMRI) study found that women with PCOS showed more activation than controls within some brain regions during a memory task, but the difference between the groups disappeared after antiandrogenic (AA) treatment (18). Another fMRI study by Lai (23) demonstrated that the amplitude of low-frequency fluctuation (ALFF) and functional connectivity (FC) were significantly different from some brain gyri between patients and healthy controls, and the FC between the right superior frontal gyrus (SFG.R) and right middle frontal gyrus (MFG.R) was negatively correlated with luteinizing hormone (LH) levels. Based on the above studies, we speculated that abnormal hormone levels and IR may lead to changes in brain function to induce cognitive impairment, but the potential neurobiological mechanisms of the effects on brain function and cognition need to be explored. Thus far, studies on the influence of women with PCOS have mainly focused on pathophysiology, clinical manifestation and treatment; there have been relatively few studies on brain and cognitive function.

In the current study, we applied resting-state functional magnetic resonance (rs-fMRI) to investigate the changes in brain activity at the regional and network levels. Specifically, to elucidate the differences in regional brain function between women with PCOS and healthy controls, amplitude of low-frequency fluctuation (ALFF) analysis was used to detect alterations of regional cerebral activity. As we know, the rs-fMRI signal is based on spontaneous, low-frequency (< 0.1 Hz) blood oxygen level-dependent (BOLD) fluctuations in the resting brain, which is an indirect measure of neuronal activity, through neurovascular coupling (24–26). ALFF analysis could reflect spontaneous fluctuations in the fMRI BOLD signal and could thus reveal the spontaneous neural function of the whole brain and represent the baseline activity. Up until now, ALFF analysis has been extensively applied to reveal the mechanisms of many neurodegenerative and cognitive impairment diseases, such as Alzheimer’s disease (27), Parkinson’s disease (28), schizophrenia (29), and anxiety disorder (30). Peak points of specific brain areas identified by ALFF analysis were then used as seeds for additional seed-based FC analysis. Compared with traditional seed-picking methods based on priori presumptions, the ALFF measurement could provide more scientific and accurate assessments of brain function alterations. FC mapping reveals brain function at the network level and contributes to locating the functional topography of the brain altered by injury or diseases (31). By combining ALFF and FC analyses, we hope to elucidate the latent mechanism of cognitive impairments and emotional disorders.

We hypothesized that alterations of brain function in women with PCOS may be related to the observed neuropsychological symptoms and abnormal hormones.

## Materials and Methods

### Subjects

The study was reviewed and approved by the local Medical Ethics Committee of Xinqiao Hospital (Chongqing, China) and followed the rules and regulations for a valid study. Before participating in the experiment, all patients provided signed informed consent after being informed of the purpose of the experiment, the procedures involved, and possible risks.

The study group consisted of 41 right-handed female subjects (age range: 18–35 years) with newly diagnosed PCOS at Xinqiao Hospital. The diagnosis of PCOS was based on the 2003 Rotterdam criteria (5).

The inclusion criteria were as follows: (1) being a right-handed female patient between 18 and 35 years old; (2) having an elementary school education or above; (3) having been newly diagnosed by an obstetrician-gynecologist based on the 2003 Rotterdam criteria; and (4) having agreed to participate in the study.

The exclusion criteria were as follows: (1) current or recent (last six months) hormonal treatment; (2) pregnant or breastfeeding; (3) other diseases that may cause hyperandrogenism or anovulation, such as congenital adrenal hyperplasia, androgen secreting tumor, prolactinoma, Cushing’s syndrome and thyroid disease; (4) history of craniocerebral injury, mental disorders, chronic diseases such as diabetes, hypertension, rheumatoid arthritis, cancer or other factors that may affect cognition; and (5) contraindications to MRI examinations such as a pacemaker, internal metal and coronary artery stenting.

Forty-one right-handed female healthy controls matched by age and education (32) were enrolled by the advertisement. All controls were 18 to 35 years old and had regular menstrual cycles (28–30 days). Controls with a personal history of diabetes or hypertension and a family history of PCOS were excluded. Physical examination and hormone detection were performed to determine the health status of control participants.

Two neuroradiologists with more than five years of working experience examined T_1_MR images to rule out gross neuroanatomical abnormalities. In addition, participants with head movements in the scanner that exceeded 2.0 mm or 2.0° were also excluded.

### Experimental Design

Our study was prospective, as we aimed to combine fMRI analysis with cognitive function tests to elucidate the latent mechanism of cognitive impairments and emotional disorders in women with PCOS and to further to study brain activity and cognitive function after treatment or hormonotherapy. The current study was the first stage of our research.

All enrolled subjects initially underwent an MR scan after a rest period lasting at least ten minutes. Blood samples were collected after overnight fasting on the 2–5 days of the menstrual cycle (33). Blood samples were collected for the assessment of the levels of sex hormones, blood lipids, fasting serum insulin, and fasting blood glucose. Finally, all the subjects were evaluated by neuropsychological assessment scales and cognitive function tests.

The main hormonal assessment kits were as follows: testosterone (2nd Generation Testosterone Reagent Kit, 0.15-64.57nmol/L), LH (ARCHITECT LH Reagent Kit, 10–70 mIU/ml), prolactin (ARCHITECT Prolactin Reagent Kit, 0.65–185.74 ng/ml), follicle stimulating hormone (ARCHITECT FSH Reagent Kit, 0.46–120.45 mIU/ml), estradiol (ARCHITECT Estradiol Reagent Kit, 10–1,000 pg/ml), and insulin (Insulin Reagent Kit, 1.0–300.0 μU/ml). All of these kits were produced by the Abbott Ireland Diagnostics Division, and samples were analyzed on the Abbott i2000 instrument.

### Structural and Functional Image Acquisition

All MRI data were obtained on a 3T GE MRI system using a brain-specific standard 8-channel head coil. Before scanning, all subjects were asked to close their eyes, not to think about specific things, not to move and to avoid falling asleep during the scan. Compliance was confirmed by the subjects after the scan, and those who were not compliant were rescanned. High-resolution T1-weighted images, which were obtained as an anatomical reference, were collected using a 3D-fast spoiled gradient-echo (FSPGR) sequence with the following parameters: repetition time (TR) = 450 ms, echo time (TE) = 2.8 ms, flip angle = 15°, field of view (FOV) = 240×240 mm^2^, matrix = 256×256, number of slices = 108, slice thickness = 1.6 mm, gap = 0 mm, and isotropic voxel size = 1.6×1.6×1.6 mm³. Blood oxygen level-dependent (BOLD) fMRI was collected using an echo-planar imaging (EPI) sequence with the following parameters: TR = 2,300 ms, TE = 30 ms, flip angle = 90°, FOV = 240×240 mm^2^, matrix = 64×64, number of slices = 34, slice thickness = 5 mm, gap = 0 mm, and isotropic voxel size = 3×3×3 mm³.

### Neuropsychological and Clinical Symptom Assessment

Each subject was required to complete a series of relevant neuropsychological and clinical symptom assessment scales. The Self-Rating Anxiety Scale (SAS, a 20-item self-report assessment designed to measure anxiety levels) and the Beck Depression Inventory-II (BDI-II, a 21-question multiple choice self-report inventory that evaluates depression levels) were used to measure differences in mood between the two groups. The Pittsburgh Sleep Quality Index (PSQI) was used to evaluate the sleep quality of all subjects.

### Cognitive Function Tests

For all subjects, three tasks based on the E-prime program were used to test cognitive function in different cognitive domains that included working memory, attention, and executive function. The two-back task was used to test working memory (34). The Stroop Color Word Test was used to assess executive function (35). The Attention Network Task (ANT) accurately measured the function of the alerting, orienting, and executive control networks (36). At the end of all tests, the accuracy and average reaction time (RT) of the answers were analyzed.

### rs-fMRI Data Processing

Based on the MATLAB 8.2.0.701 (R2013b) platform, the Data Processing Assistant for Resting-State fMRI Advanced Edition (http://rfmri.org/DPASFA) was used to preprocess and analyze all of the fMRI data. Initially, MR images were converted from a DICOM format to a NIFTI format. As the magnetic field was unstable and the participants needed a certain amount of time to adapt to the noisy environment, the functional images collected during the first 10 time points were discarded. By slice-timing correction of the remaining 260 functional images, the slices acquired at different times within a repetition time were corrected for temporal shifts between slices. After being realigned for head motion, the MR images were removed if they showed head movement more than 2.0 mm or rotation greater than 2.0° in any direction. Then, the functional images and the T1-weighted structural images were coregistered. The entire brain was segmented into gray matter (GM), which was used in the subsequent spatial normalization, WM and cerebrospinal fluid (CSF). Next, the Friston 24-parameter model, head motion scrubbing with a threshold of 0.5 mm, and the noisy signals of the WM and CSF were chosen for the nuisance covariate regression to reduce noise. There has been controversy regarding retaining the whole brain signal (37); nevertheless, we chose to retain the whole brain signal. Then, the GM was spatially normalized to the standard Montreal Neurological Institute (MNI) template, and voxel size was set to 3 mm × 3 mm× 3 mm for resampling. To reduce the spatial differences between subjects, a Gaussian kernel was set as 6-mm full-width at half-maximum (FWHM) for spatial smoothing. Finally, detrending was performed to remove the linear trends. The bandpass filter was set at 0.01–0.08 Hz.

### ALFF and FC Calculations

A preprocessed image with bandpass filtering (0.01 < f < 0.08 Hz), which reduced low-frequency drift and high-frequency breathing and heart noise, was used to calculate the whole brain ALFF in each subject. The voxel wise ALFF map at the subject level was converted to a z-score map by subtracting the mean ALFF of the entire brain and dividing that value by the standard deviation (38). With the rs-fMRI data analysis toolkit (REST v1.8) (http://www.restfmri.net/forum/REST_V1.8), FC was calculated in MATLAB (39). The peak point from ALFF analysis was selected as the coordinate for those regions of interest (ROIs), and the radius was set to 6 mm. The seed-based FC map between the time course of the seed region and the time series of all voxels in the global brain was calculated by Pearson correlation analysis. Finally, in all maps from the previous statistical analysis, Fisher’s r-to-z transformation was applied.

### Statistical Analysis

We performed statistical analyses by using SPSS 24.0 (SPSS Inc., Chicago, IL). To analyze the differences in the demographic data, scores on the neuropsychological scales, accuracy and RTs for the ANT, Stroop, and two-back tasks between the two groups, independent sample t-tests were used. In addition, if the variable did not show a normal distribution, we performed the Mann-Whitney rank test for analysis. Statistical significance was set to *p <*0.05.

To investigate the difference in ALFF values and FC between 41 women with PCOS and 41 healthy controls, SPM 8 software was used to perform a two-sample t-test between the two groups. The level of education and age were regressed as covariables in the calculation. The topological false discovery rate (FDR), which is a rigid approach, was used to correct for multiple comparisons in the whole brain (40). For the above analyses, we set the voxel-level threshold at *p* = 0.001 for the FDR-corrected *p* < 0.05. After regression of the influence of age and educational level, Pearson’s correlation coefficients were performed by SPSS software to evaluate the relationship between clinical indexes, neuropsychological results, ALFF values and FC strength in women with PCOS. Statistical significance was set at *p <*0.05.

## Results

### Demographic and Clinical Characteristics

The demographic characteristics, neuropsychological symptoms and clinical symptoms of the PCOS group and the healthy control group are shown in **Table 1**. There were no statistically significant differences in age (p = 0.15) or educational level (p = 0.312) and accuracy rate of Stroop test (p = 0.066) between the two groups. The PCOS group had significantly higher scores on the BDI-II (p < 0.001), SAS (p < 0.001), and PSQI (p = 0.001) than the healthy control group, and the cognition test results showed that the PCOS group had longer alerting times (p = 0.026), executive control times (p = 0.021), two-back RTs (p = 0.001) and Stroop test RTs (p = 0.001) but lower accuracy rate in the Two-back test (p < 0.001) than the healthy control group. The T, LH, FINS, HOMA2-IR, and triglyceride (TG) levels of the PCOS group were significantly higher (p < 0.001) than those of the healthy control group. Fourteen women with PCOS with a HOMA2-IR score greater than 1.7 were thought to have IR (41, 42), constituting about one-third of the total.

**Table 1:** Demographic, clinical characteristics, mood, and cognitive performance data.

Characteristic	PCOS women	Healthy control women	*p*-value
*n*	41	41	
Age(years)	25.29 ± 3.15	26.22 ± 2.59	0.15^a^
Edu(years)	14.43 ± 1.96	14.93 ± 2.36	0.312^a^
BMI(kg/m^2^)	24.62 ± 4.88	20.31 ± 1.81	<0.001^a^
T(nmol/L)	2.89 ± 1.14	1.71 ± 0.33	<0.001^b^
FSH(mIU/ml)	5.02 ± 1.47	5.05 ± 1.44	0.932^a^
LH(mIU/ml)	11.90 ± 7.78	4.28 ± 1.19	<0.001^b^
TG(mmol/L)	1.60 ± 1.04	0.94 ± 0.35	<0.001^a^
FINS(uU/ml)	12.17 ± 7.67	6.25 ± 1.77	<0.001^b^
HOMA2-IR*	1.53 ± 0.95	0.79 ± 0.22	<0.001^b^
BDI-II	13.95 ± 10.43	5.51 ± 6.18	<0.001^b^
SAS	45.15 ± 8.21	35.98 ± 8.10	<0.001^a^
PSQI	7.19 ± 3.10	4.93 ± 2.76	0.001^a^
**ANT**			
Alerting(ms)	52.16 ± 23.15	40.25 ± 24.53	0.026^a^
Orienting(ms)	26.18 ± 22.83	35.31 ± 26.59	0.293^b^
Executive controls(ms)	115.79 ± 48.05	89.84 ± 29.24	0.021^b^
**Two-Back**			
ACC	0.70 ± 0.14	0.82 ± 0.13	<0.001^b^
RT(ms)	748.44 ± 114.63	673.36 ± 81.46	0.001^a^
**Stroop Test**			
ACC	0.85 ± 0.15	0.91 ± 0.06	0.066^b^
RT(ms)	768.97 ± 136.82	679.47 ± 98.72	0.001^a^

Edu, education; BMI, Body Mass Index; T, Testosterone; FSH, follicle-stimulating hormone; LH, luteinizing hormone; TG, Triglyceride; FINS, fasting insulin; HOMA2-IR, homeostatic model assessment index for insulin resistance; BDI-II, Beck depression inventory II; SAS, self-rating anxiety scale; PSQI, Pittsburgh Sleep Quality Index; ANT, Attention Network Task; ACC, accuracy rating; RT, reaction time.

a Two-sample independent t-test (41 women with PCOS vs 41 healthy control women).

b Mann-Whitney U test (41 women with PCOS vs 41 healthy control women).

*HOMA2-IR was calculated by HOMA2 Calculator v2.2.3 (https://www.dtu.ox.ac.uk/homacalculator/).

### ALFF Analysis

**Figure 1:**
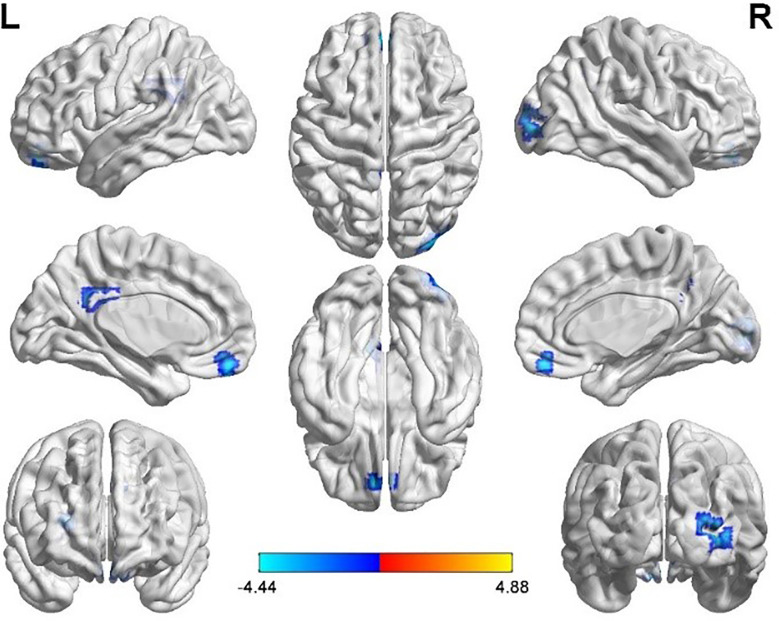
Significantly increased (red) and decreased (blue) ALFF values in women with polycystic ovary syndrome compared with controls (the peak level threshold p = 0.001; cluster level FDR-corrected, p < 0.05). The color bar represents the t-value of the two-sample t-test between the two groups.

Several related brain regions showed significant differences in ALFF in women with PCOS compared with the healthy controls, and the results are summarized in **Table 2** (FDR-corrected, voxel-level threshold p = 0.001 and cluster-level threshold p < 0.05; **Figure 1**). All ALFF values in the left middle frontal gyrus (MFG.L), left posterior cingulate gyrus (PCG.L) and right middle occipital gyrus (MOG.R) were lower in the PCOS group than in the healthy control group. The above peak points were selected as seed points for further FC analysis.

**Table 2:** ALFF alterations between the two groups.

Brain region	BA	MNI peak point coordinates			t-value	Voxels
		X	Y	Z		
MFG.L	11	-6	51	-18	-4.44	80
PCG.L	31	-9	-45	27	-4.88	83
MOG.R	18	30	-96	9	-4.06	93

ALFF, amplitude of low-frequency fluctuations; MNI, Montreal Neurological Institute; BA, Brodmann area; MFG.L, left middle frontal gyrus; PCG.L, left posterior cingulate gyrus; MOG.R, right middle occipital gyrus; SFG.R, right superior frontal gyrus. False discovery rate (FDR)-corrected, p < 0.05.

### FC Analysis

The three peak points in the MFG.L, PCG.L, and MOG.R were set as the seed points, and two-sample t-tests were carried out in SPM8 to determine the significant differences in FC strength between these seed points and the whole brain (**Tables 2** and **3**).

**Table 3:** FC alterations between the two groups.

Connected Regions	BA	Peak Areas	MNI coordinates			t-value	Voxels
			X	Y	Z		
Seed Point (-6 51 -18)							
	45	IFG.L	-57	15	6	5.10	107
	40	IPL.R	48	-33	45	4.23	118
Seed Point (-9 -45 27)							
	7	MOG.L	-30	-63	30	6.11	168
	6	MFG.L	-24	6	51	5.33	261
	6	MFG.R	33	6	48	5.31	166
	6	SFGmed.L	3	30	39	4.74	98
	46	IFGtriang.L	-36	39	6	4.85	274
Seed Point (30 -96 9)							
	19	IOG.R	42	-87	3	-5.23	129

FC, functional connectivity; MNI, Montreal Neurological Institute; BA, Brodmann area; IFG.L, left inferior frontal gyrus; IPL.R, right inferior parietal lobe; MOG.L, left middle occipital gyrus; MFG.L/MFG.R, left/right middle frontal gyrus; SFGmed.R, right superior frontal gyrus, medial; IFGtriang.L, left inferior frontal gyrus, triangular part; IOG.R, right inferior occipital gyrus. FDR-corrected, p < 0.05.

**Figure 2:**
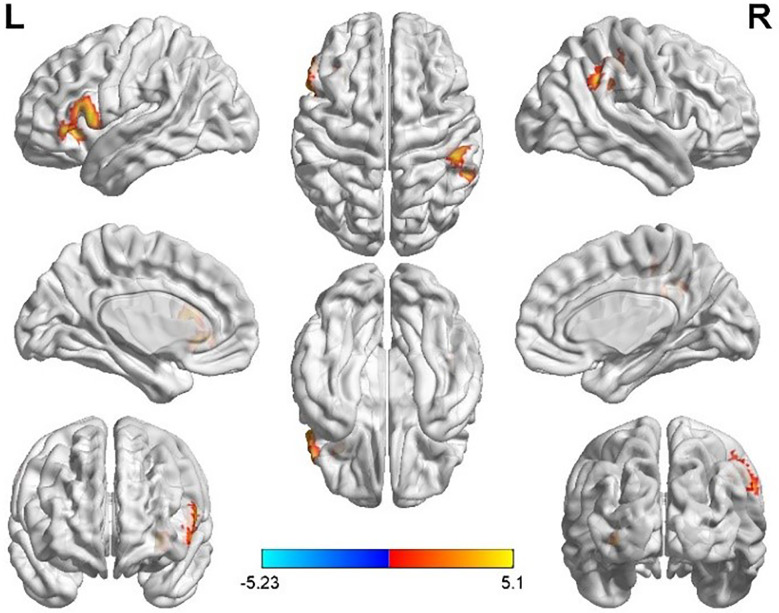
Differences in left middle frontal gyrus (MFG.L) based FC between women with polycystic ovary syndrome and controls (the peak level threshold was set at p = 0.001; cluster level FWE-corrected, p < 0.05). The FC strength was increased and marked in red.

Taking the peak point in the MFG.L (-6, 51, -18) as the first seed point, FC strength with the left inferior frontal gyrus (IFG.L) and right inferior parietal lobe (IPL.R) was enhanced (p < 0.05, FDR correction; **Figure 2**).

**Figure 3:**
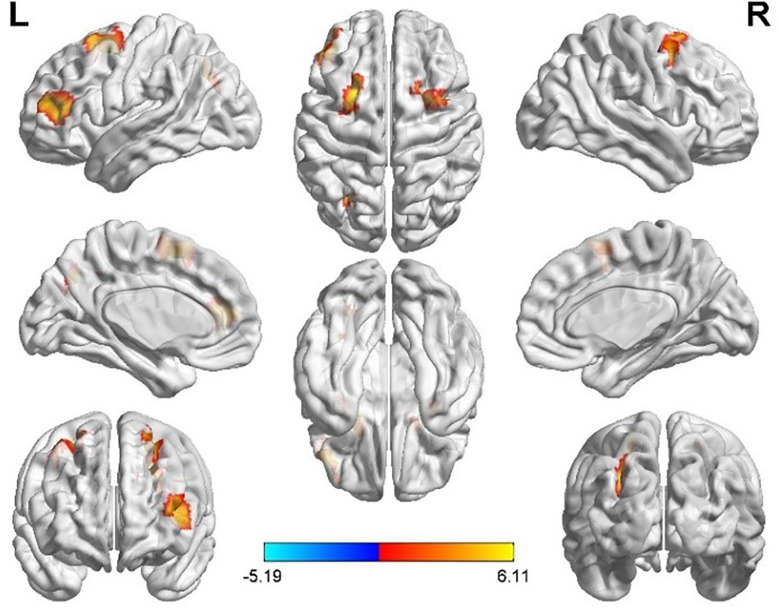
Differences in left posterior cingulate gyrus (PCG.L) based FC between women with polycystic ovary syndrome and controls (the peak level threshold was set at p = 0.001; cluster level FDR-corrected, p < 0.05). All the FC strengths were increased and marked in red.

Taking the peak point in the PCG.L (-9, -45, 27) as the second seed point, FC strength with some brain regions, including the left middle occipital gyrus (MOG.L), the bilateral middle frontal gyrus (MFG.B), the medial part of the superior frontal gyrus (SFGmed.L), and the triangular part left inferior frontal gyrus, (IFGtriang.L) were significantly enhanced (p < 0.05, FDR correction; **Figure 3**).

**Figure 4:**
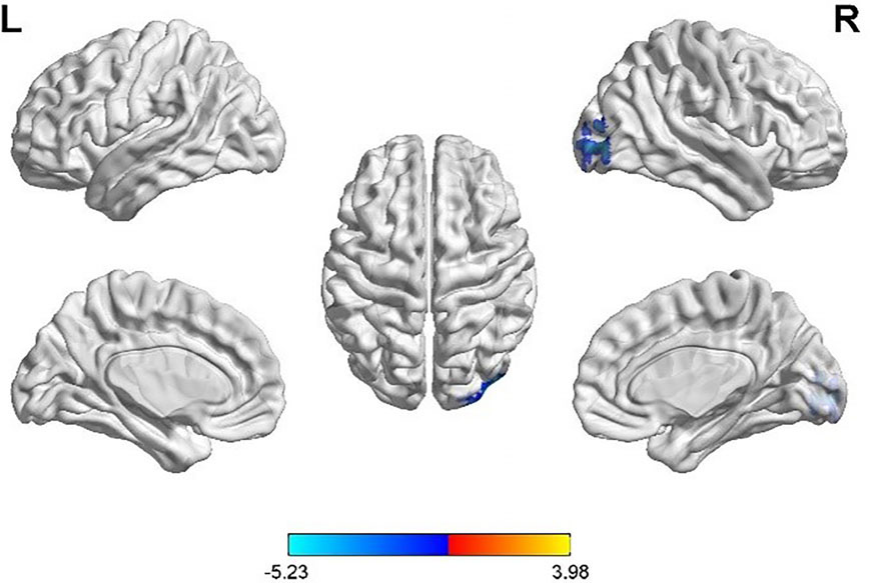
Differences in right middle occipital gyrus (MOG.R) based FC between women with polycystic ovary syndrome and controls (the peak level threshold was set at p = 0.001; cluster level FDR-corrected, p < 0.05). The FC strength was decreased and marked in blue.

Taking the peak point in the MOG.R (30, -96, 9) as the third seed point, FC strength with the right inferior occipital gyrus (IOG.R) was decreased (p < 0.05, FDR correction; **Figure 4**).

### Correlations Between Neuropsychological Data and Abnormal ALFF Values and FC Strength

**Figure 5:**
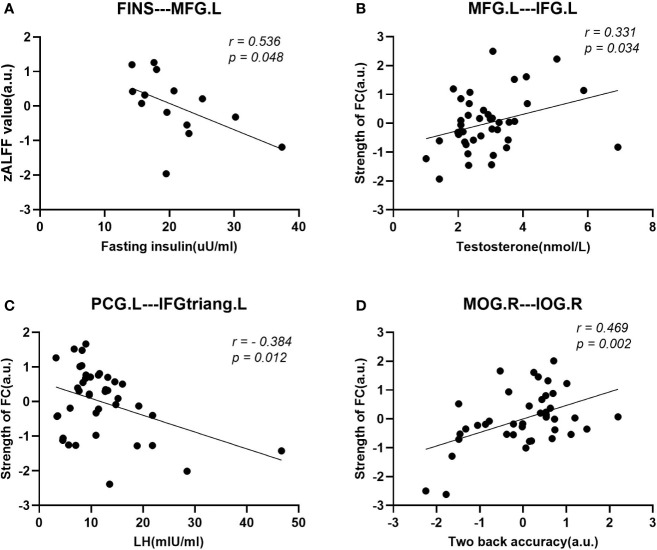
Scatter diagrams showing the significant correlations between aberrant ALFF values, FC strengths, clinical data, and neuropsychological assessments of women with PCOS in **(A–D)**. **(A)** The decreased ALFF value in the MFG.L was negatively correlated with the fasting insulin (FINS) within insulin resistant subjects. **(B)** The enhanced FC strength between the MFG.L and IFG.L was positively correlated with the plasma T level. **(C)** The enhanced FC strength between the PCG. L and the IFGtriang.L was negatively correlated with the luteinizing hormone (LH). **(D)** The accuracy rate (ACC) in the two-back task was positively correlated with the decreased FC strength between the MOG.R and the IOG.R.

After regressing age and educational level, we performed Pearson’s correlation analyses among the women with PCOS. First, the ALFF value in the MFG.L was negatively correlated with serum FINS (r = 0.536, p = 0.048) within subjects with IR, while no significant correlation was found for subjects without IR. Second, the enhanced FC strength between the MFG.L and the IFG.L was positively correlated with T (r = 0.331, p = 0.034) and enhanced FC strength between the PCG.L and the IFGtriang.L was negatively correlated with LH (r = -0.384, p= 0.012). Finally, the FC strength between the MOG.R and IOG.R was positively correlated with the accuracy of the two-back tasks (r = 0.469, p = 0.002). The results of these correlation analyses are shown in **Figure 5**.

## Discussion

This study, by using rs-fMRI in women with PCOS, not only revealed regional abnormal brain activity in a resting state but also analyzed whole-brain FC. Here, we speculate on the underlying neural mechanisms of cognitive dysfunction in these patients. We observed three aspects of altered functionality: (1) decreased ALFF values in the MFG.L, PCG.L and MOG.R in women with PCOS compared with healthy controls; (2) increased FC strength between the MFG.L and IFG.L and IPL.R based on seed-based FC analyses; increased FC strength between PCG.L and other brain regions, including the MOG.L, bilateral MFG, SFGmed.L and IFGtriang.L; and decreased FC strength between the MOG.R and IOG.R; and (3) impaired emotional and cognitive functions, assessed by neuropsychological scales and cognitive function tasks, in women with PCOS compared with healthy controls. As discussed below, our results illuminate the potential mechanisms underlying the cognitive impairments and neuropsychological complications observed in PCOS patients.

The aberrant brain activity of women with PCOS might be related to hormone disorders. Previous studies have shown that changes in brain activity and cognitive function in women with PCOS might be associated with androgen excess, especially high circulating levels of T. Women with PCOS demonstrated impaired performance regarding RT and word recognition tasks, but performance could be improved with AA medication compared with the PCOS group without AA treatment (18). Another similar study found that women with PCOS had higher free T levels and worse performance on tests of verbal fluency, verbal memory, manual dexterity, and visuospatial working memory than healthy women (19). Soleman et al. found that women with PCOS showed increased activation within the right superior parietal lobe (SPL.R) and the IPL.R than the control women during working memory tasks; however, after AA treatment, the difference between the groups disappeared (33). All these studies indicated that androgen excess may lead to cognitive impairment, such as those evident in verbal, motor, and visual-spatial abilities. In this study, we found enhanced FC strength between the MFG.L and IFG.L was positively correlated with the plasma T levels. This result indicated that high serum T levels might lead to changes in brain activity.

The decrease in the ALFF value in the MFG.L might be involved in the neuropathological mechanisms underlying working memory function impairments. The prefrontal cortex (PFC) has been reported to be associated with many cognitive functions (43), such as execution, emotion and working memory (44, 45), but the function of the PFC is asymmetric, with the left PFC associated with positive emotions and the right PFC associated with negative emotions (46). Some studies demonstrated that the left lateral PFC played an important role in two-back task performance, and decreased activity in the left PFC was linked with depression (47). In our study, the PCOS group showed higher two-back RTs but showed lower accuracy rates compared with the control group. In addition, the PCOS group demonstrated higher scores on the BDI-II. By using Pearson’s correlation analysis, we found that the decreased ALFF value in the MFG.L was negatively correlated with the plasma FINS level within insulin-resistant women, but we did not find this correlation for women without IR. According to the above results, we think that the decreased ALFF value in the MFG.L might be caused by IR, and it will further lead to cognitive impairment.

The posterior cingulate cortex (PCC) belongs to the cortical part of the limbic system, is a central part of the default mode network (DMN), plays an important role in the emotional circuit and is involved in processes such as emotion and self-evaluation, which are closely associated with depressive symptoms (48). An rs-fMRI study (49) found aberrant resting-state FC from the PCC to other brain regions, especially to the MTG.R, was associated with IR and cognitive performance. We found that the PCOS group had significantly higher scores on the BDI-II and SAS, which might be incurred by the decrease in the ALFF value in the PCG.L. Pearson correlation analysis demonstrated that FC strength between the PCG.L and the IFGtriang.L was negatively correlated with the LH. Lai W et al. found that the FC between the SFG.R and MFG.R was negatively associated with the plasma LH level, as well as the LH/FSH ratio (23). The above results indicate that high serum LH levels might incur changes in brain activity, further leading to cognitive function impairment.

The occipital lobe is the visual cortex center, and occipital lobe disease damage not only causes visual impairment but also memory deficits, motor perception disorders and other symptoms. Golby et al. found that altered activation in the occipital gyrus was associated with damaged visual memory (50). A previous fMRI study of Cushing’s syndrome (CS) revealed functional brain responses in the IOG.R was correlated with episodic-memory encoding (51). This study showed that the ALFF value in the MOG.R was decreased, and the FC strength between MOG.R with IOG.R was also decreased. Pearson’s correlational analysis revealed decreased FC strength between the MOG.R and the IOG.R was positively correlated with the working memory task accuracy. These results suggested that the worse the FC between MOG.R and IOG.R was, the worse the memory was in women with PCOS.

Increasing evidence suggests that androgen excess facilitates IR and metabolic dysfunction in women with PCOS. In addition, IR, hyperinsulinemia and LH could enhance androgen secretion by the ovaries and reduce the synthesis of sex hormone binding globulin (SHBG) in the liver (6, 52). Such interactions lead to elevated insulin levels and IR. Numerous clinical studies have shown that IR is a risk factor for cognitive dysfunction (53–55). Ekblad et al. found that people with high plasma FINS and IR represent poorer verbal fluency performance compared with controls (56). Research by Jarrett et al. indicated that women with PCOS have worse visuospatial ability than healthy control subjects, and decreased visuospatial ability was linked to higher levels of hemoglobin A1c (57). Another study showed that women with PCOS had a lower cerebral metabolic rate of glucose (CMRglu) in specific brain regions, such as the frontal, parietal and temporal cortices, and the CMRglu in the frontal, parietal and temporal cortices was significantly negatively correlated with HOMA2-IR (20). Based on the above view, the activity and structure of particular brain regions might be damaged by high concentrations of insulin and IR. In this study, the PCOS group had obviously higher FINS levels and HOMA2-IR scores than those of the control group, and women with PCOS showed decreased ALFF values in the MFG.L, PCG.L and MOG.R, which might be associated with IR and a decrease in CMRglu in these brain areas. Lansdown et al. found that insulin sensitivity was associated with brain BOLD signal changes in women with PCOS, and the difference disappeared after adjusting for insulin sensitivity (58). Ekblad et al. (56) also demonstrated that higher baseline IR and plasma FINS could predict poor verbal fluency among the adult population in the future. In this study, the plasma FINS level of women with IR was negatively correlated with the ALFF value in the MFG.L. In summary, we think that abnormal brain activity in women with PCOS might be associated with high IR and serum FINS levels, and treatment with IR might be beneficial to women with PCOS and reduce cognitive function decline in their life from that point forward.

Our research also has some limitations. First, the sample size of this study was relatively small (59, 60), and the body mass index (BMI) between the two groups was not strictly matched. In addition, the inclusion criteria for PCOS were mainly based on the 2003 Rotterdam criteria, and different phenotypes (61–63) were not taken into consideration. Second, the different social experiences and occupations were uncontrollable factors that may have influenced the abnormal brain activity that we observed. We tried to eliminate those individuals with large differences through pre-experimental screening to minimize the impact of these factors on the results. Finally, rs-fMRI and cognitive function assessment of the PCOS group were not conducted after AA and treatment of IR due to the limited time that the women with PCOS were available. The effect of plasma high free T and LH levels and high IR on women with PCOS needs to be further explored in future studies.

## Conclusion

In conclusion, rs-fMRI was used in our study to observe changes in regional and network levels of brain function in women with PCOS, which have provided insight into the mechanisms of neurobiological changes in women with PCOS. The results showed that abnormal ALFF and FC were mainly located in the MFG.L, PCG.L, MOG.R, and IFG.L, IPL.R, MOG.L, bilateral MFG, SFGmed.L, IFGtriang.L, and IOG.R and were related to disrupted cognitive function and emotional changes. Thus, our findings might have revealed the mechanisms of brain dysfunction associated with T, LH and FINS, and patterns of change in ALFF values and FC may be used as neuroimaging biomarkers in clinical practice. Increasing insulin sensitivity and reducing high circulating levels of LH and T will be beneficial to women with PCOS.

## Data Availability Statement

The raw data supporting the conclusions of this article will be made available by the authors, without undue reservation.

## Ethics Statement

The studies involving human participants were reviewed and approved by Medical Ethics Committee of Xinqiao Hospital (Chongqing, China). The patients/participants provided their written informed consent to participate in this study.

## Author Contributions

GL and JH contributed to performing the experiments, analyzing data, and writing of the manuscript, and as the first author. LW designed the experiment. SZ and WF contributed to the recruiting subjects and the analyzing data. GW contributed to revising the manuscript. DZ is the guarantor of this study and is fully accessible to all data in the study and is responsible for the integrity of the data and the accuracy of the data analysis. All authors contributed to the article and approved the submitted version.

## Funding

This study was funded by the Army Medical University (2016YLC08). The funder had no role in the study design, the data collection, the analysis and interpretation, the writing of the manuscript or the decision to submit this article for publication.

## Conflict of Interest

The authors declare that the research was conducted in the absence of any commercial or financial relationships that could be construed as a potential conflict of interest.
